# Exploring the role of mitochondrial antiviral signaling protein in cardiac diseases

**DOI:** 10.3389/fimmu.2025.1540774

**Published:** 2025-02-18

**Authors:** Yuying Qi, Jie Yin, Weiwei Xia, Shiwei Yang

**Affiliations:** ^1^ Department of Cardiology, Children’s Hospital of Nanjing Medical University, Nanjing, China; ^2^ Department of Clinical Laboratory, Children’s Hospital of Nanjing Medical University, Nanjing, China

**Keywords:** MAVS, NLRP3, heart disease, innate immune, inflammation

## Abstract

Mitochondrial antiviral signaling (MAVS) was first discovered as an activator of NF-κB and IRF3 in response to viral infection in 2005. As a key innate immune adapter that acts as an ‘on/off’ switch in immune signaling against most RNA viruses. Upon interaction with RIG-I, MAVS aggregates to activate downstream signaling pathway. The MAVS gene, located on chromosome 20p13, encodes a 540-amino acid protein that located in the outer membrane of mitochondria. MAVS protein was ubiquitously expressed with higher levels in heart, skeletal muscle, liver, placenta and peripheral blood leukocytes. Recent studies have reported MAVS to be associated with various conditions including cancers, systemic lupus erythematosus, kidney disease, and cardiovascular disease. This article provides a comprehensive summary and description of MAVS research in cardiac disease, encompassing structure, expression, protein-protein interactions, modifications, as well as the role of MAVS in heart disease. It is aimed to establish a scientific foundation for the identification of potential therapeutic target.

## 1 Introduction

The mitochondrial antiviral signaling protein (MAVS) was initially discovered as a crucial molecule of antiviral innate immunity and is also referred to as IPS-1, VISA and Cardif (1–4) in 2005. It contains an N-terminal caspase activation and recruitment domain (CARD), a central proline-rich region (PRR) and a C-terminal transmembrane domain (TM). It plays a central role in regulating the complex processes that result in antiviral or inflammatory responses (5). Located ubiquitously on the outer mitochondrial membrane, peroxisomes and endoplasmic reticulum (6). MAVS acts as an articulatory protein. Retinoic acid-inducible gene I (RIG-I, also known as DExD/H-box helicase 58, DDX58, belonging to the RLRs family, detects exogenous RNAs, including viral RNAs. During viral infection, RIG-I identifies viral RNA, triggering the association between the CARD domains of MAVS and RIG, ultimately leading to the formation of MAVS aggregates. Subsequently, MAVS interacts with TNF receptor-associated factor 3 (TRAF 3) to recruit downstream IRF 3 and NF-κB activated kinases, triggering the innate immune response (5). MAVS has been recognized as a pivotal regulatory target for viruses and hosts, due to its dual function in immune homeostasis and antiviral signaling. Beyond its well-established role in antiviral defense, MAVS has emerged as a key effector in various physiological and metabolic processes. Recent studies demonstrate that MAVS is implicated in responses to bacterial and parasitic infections, autoimmune diseases, cancer advancement, kidney diseases and cardiovascular diseases (7–10). While MAVS is well-known for its role in antiviral immunity and various other immune responses, some studies have documented its involvement in viral myocarditis, cardiac insufficiency and CVD (11–13). The pathophysiological mechanisms of cardiovascular disease are complex and involve many pathological processes, including endothelial dysfunction, imbalance of calcium regulatory homeostasis, abnormal cardiac autophagy, autonomic dysfunction, metabolic reprogramming, iron imbalance, oxidative stress, inflammation, impaired mitochondrial dynamics, impaired mitochondrial autophagy, imbalance of NO synthesis. In the study of the molecular mechanisms of CVD shows a growing focus on the role of MAVS, crucial for the spontaneous high basal expression of IFN-β in the heart (14). Research has revealed that both partial or complete MAVS deficiency in mice leads to decreased cardiac function and enlarged hearts in mice due to disruptions in mitochondrial function, energy production, and lipid metabolism (11). Therefore, this article offers a brief summary of the molecular biology, protein interactions, modifications and research progress of MAVS and elucidating role of MAVS in cardiac disease to establish a scientific basis for therapeutic intervention.

## 2 Structure and expression of MAVS

**Figure 1:**
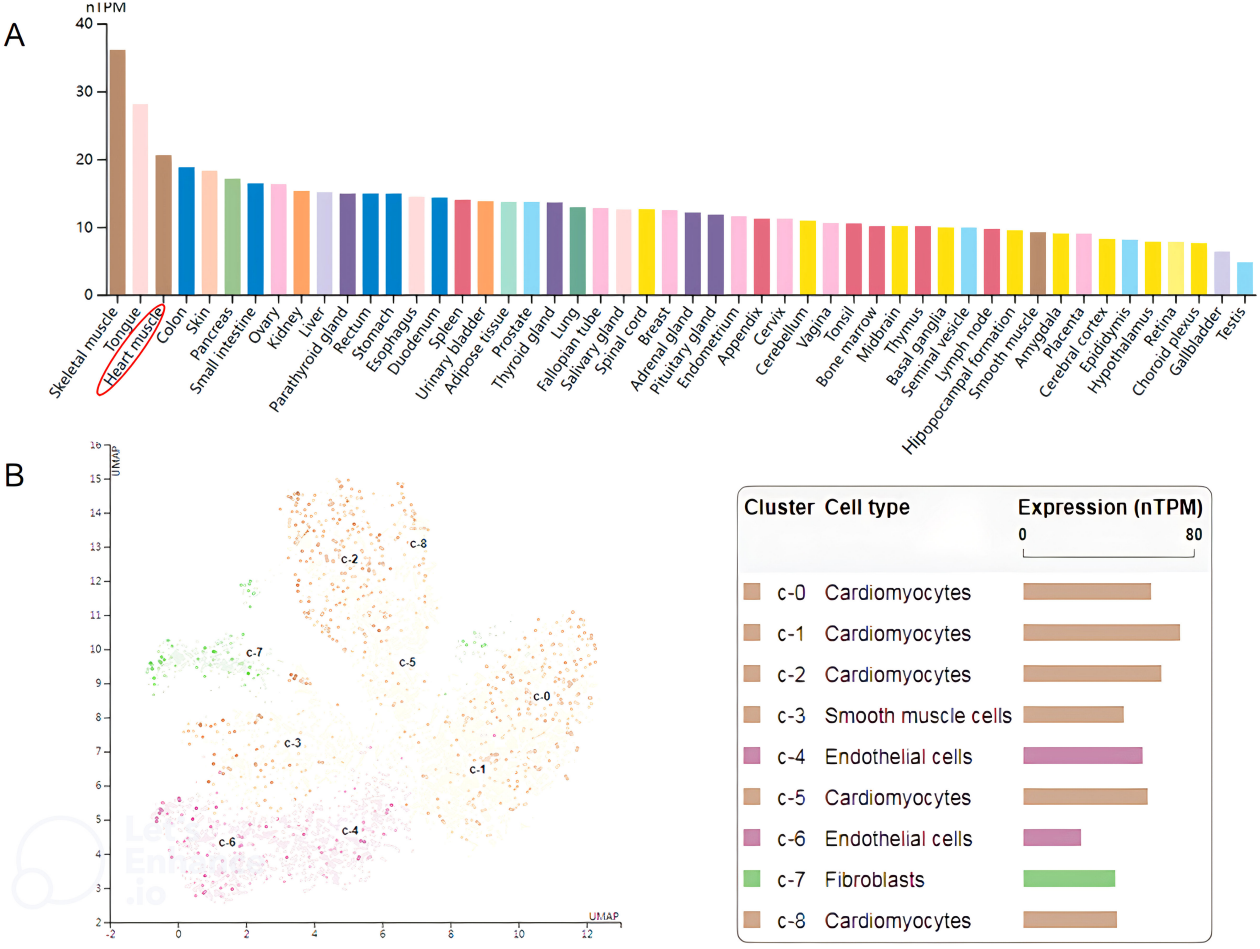
**(A)** Relative MAVS expression at the mRNA level in different human tissues. The data shown were derived from The Human Protein Atlas (http://www.proteinatlas.org/). **(B)** The expression of MAVS is enriched in cardiomyocytes. The data shown were derived from The Human Protein Atlas (http://www.proteinatlas.org/).

The human *mavs* gene locates on chromosome 20 and shares approximately half amino acid identity with its mouse counterpart (15). The full−length of *mavs* mRNA consists of 2912 bp. According to the Human Protein Atlas (http://www.protein-atlas.org/), the highest mRNA levels of MAVS are found in the skeletal muscle, tongue and heart muscle, whereas the lowest levels are found in choroid plexus, gallbladder and testis. The mRNA level of MAVS in different human tissues, is illustrated in **Figure 1A**. We furtherly analyzed the expression of MAVS mRNA levels in different single cell type of heart muscle in the website (http://www.protein-atlas.org/), and found that the highest MAVS mRNA levels were in cardiomyocytes and lowest levels in endothelial cells (**Figure 1B**).

**Figure 2:**
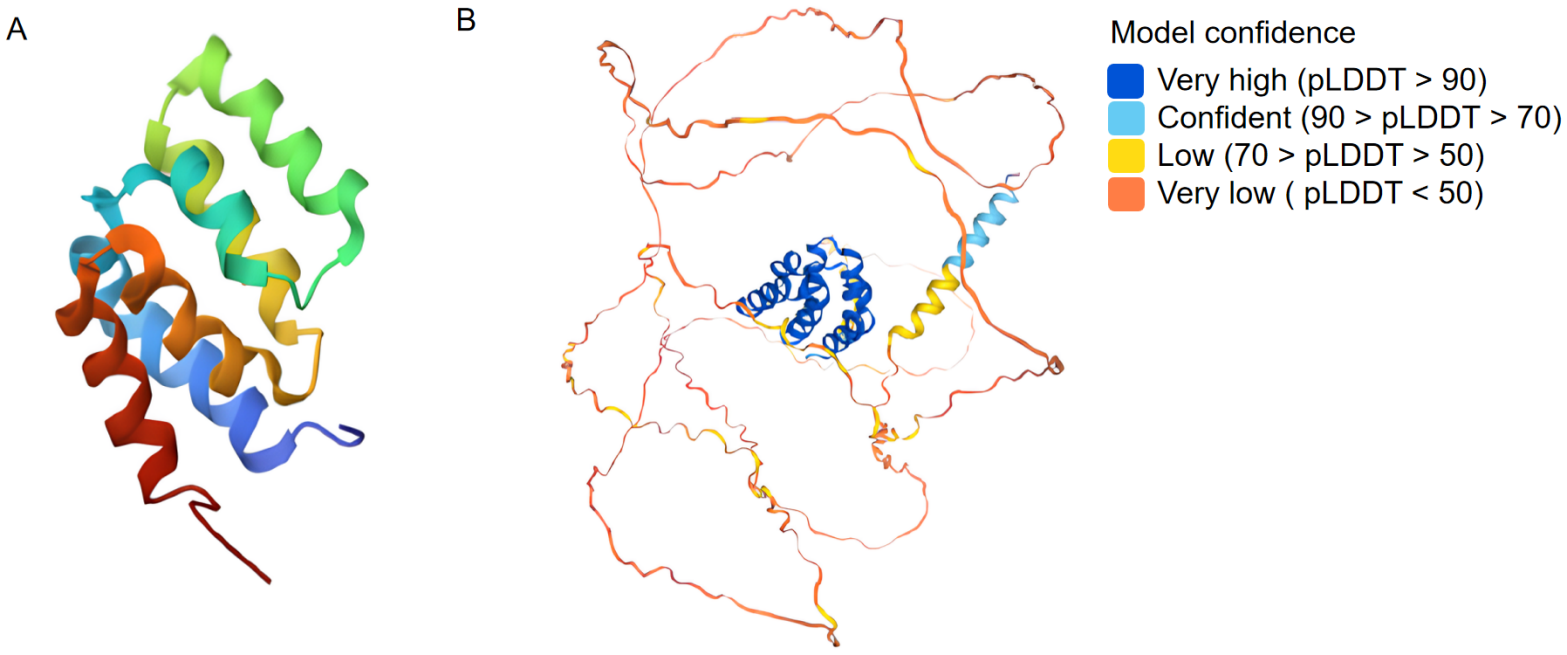
Three dimensional structures from PDB (**A**: representative) and AlphaFold (**B**: predicted) for MAVS.

MAVS is composed of three primary structural domains: the N-terminal cysteine caspase recruitment and activation domain (CARD), the internal proline-rich region and the C-terminal transmembrane domain (TM) (16). The N-terminal CARD of MAVS plays a crucial role in facilitating protein-protein interactions, establishing a significant foundation for the assembly of signaling complexes crucial for antiviral response mechanisms. The CARD domain interacts with RIG-I and MDA5 during viral infection or upon exposure to exogenous nucleic acids. Subsequently, the activated MAVS complex recruits IKK and TBK1/IKKi complexes to induce transcriptional expression of type I interferon by facilitating the nuclear translocation of NF-κB and IRF3/IRF7 transcription factors. This process triggers innate antiviral responses (17). The deletion of the CARD-like structural domain in MAVS abolishes its signaling function, converting it into a dominant-negative mutant that inhibits interferon-induced responses (3). The C-terminal transmembrane domain of MAVS functions to anchor MAVS to the mitochondrial membrane, aiding in MAVS signaling and suggesting that mitochondria serve as a functional platform for innate antiviral signaling (3, 18, 19). PRR (Proline-rich domain) structural domains are proline-rich protein motifs that bind to members of the tumor necrosis factor receptor-associated factor (TRAF) family, including: TRAF2, TRAF3, TRAF5 and TRAF6, thereby mediating downstream signal transduction (20). We also presented the 3D structures of MAVS from PDB (representative) and AlphaFold (predicted) (**Figure 2**).

## 3 The protein-protein interactions and modification of MAVS

**Figure 3:**
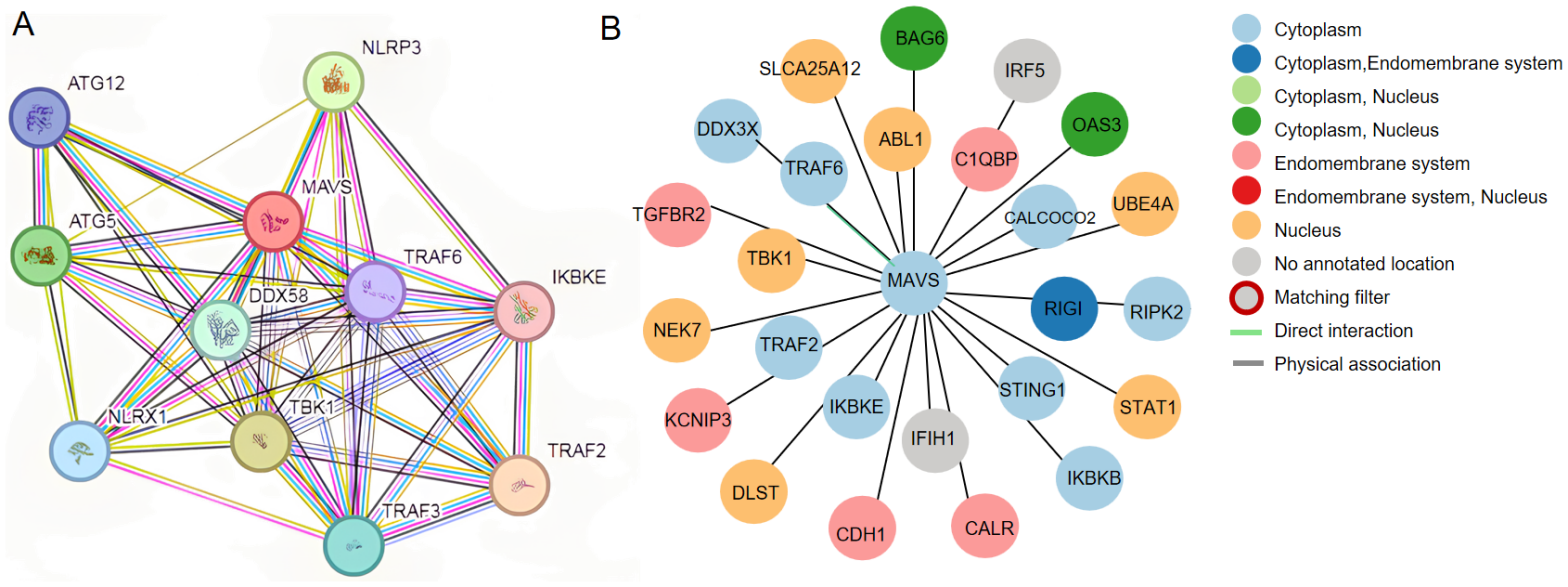
**(A)** Potential interplay between MAVS, DDX58(RIG-I) and NLRP3. The data shown were from the STRING database (www.string-db.org). **(B)** Prediction of protein interactions related to MAVS based on Human Protein Atlas (http://www.proteinatlas.org/).

Protein−protein interactions play vital roles in cellular biological processes. MAVS is known to interact with multiple proteins to activate downstream signaling pathways, including proteins in RIG-I/MAVS signaling and proteins that regulate RIG-I/MAVS signaling. The potential interacted proteins are shown in **Figure 3**. Modifications of MAVS include protein ubiquitination and phosphorylation. They are discussed below.

### 3.1 Ubiquitination

Ubiquitination plays a crucial role as a post-translational modification of host proteins, which is essential for establishing an effective antiviral response (21). Regulated post-translational modifications of host proteins by regulatory ubiquitin ligases and deubiquitinating enzymes play a role in the regulation of RLR/MAVS-mediated signaling (22). There are three ubiquitylation patterns of MAVS, including K27-linked ubiquitylation, K48-linked ubiquitylation, and K63-linked ubiquitylation. Some researchers report about K27-linked ubiquitylation of MAVS, which primarily functions in the autophagic degradation of MAVS. E3 ubiquitin ligase MARCH8, RNF34 and RNF5 are recruited to catalyze K27-linked ubiquitin chains on MAVS, resulting in the autophagic degradation of MAVS and inhibit the innate immune response (23–27). However, another E3 ubiquitin ligase TRIM21 interacts with MAVS and catalyzes K27-linked polyubiquitination, promotes the recruitment of TBK1 to MAVS and positively regulates innate immune response (28, 29). K48-linked ubiquitylation has been reported to mediate the degradation of MAVS and regulation of innate antiviral immunity. Most ubiquitination enzymes mediate the K48-linked ubiquitylation of MAVS, including TRIM25, TRIM28, MARCH5, RNF5, RNF90, RNF115, RNF146, AIP4 and SUMRF1/2. Most of them target MAVS through K48-linked polyubiquitination and negatively regulated the RLR signaling pathway by degrading MAVS (30–38). While TRIM25 activates the type-I interferon signaling pathway by degrading MAVS via K48-linked polyubiquitination (39). MAVS K63-linked ubiquitylation promotes the activation of the RIG-I/MAVS signaling by enhancing MAVS aggregation. MAVS K63-linked ubiquitylation promotes the interaction of RIG-I and MAVS though their CARD domains. TRIM31 is reported to interact with MAVS and catalyze the K63-linked polyubiquitination of Lys10, Lys311 and Lys461 on MAVS, leading to enhanced cellular antiviral response (40). N4BP3 facilitates the K63-linked ubiquitination modification of MAVS and mediates the innate immune response by accelerating the interaction of MAVS and TRAF2 (41).

### 3.2 De-ubiquitylation of MAVS

Deubiquitination is the reverse reaction of the ubiquitination process. In addition to maintaining appropriate levels of MAVS protein, de-ubiquitylation also plays an important role in the innate immunological signaling. De-ubiquitinases involved in the regulation of MAVS are listed in **Table 1**, including TRIM44, USP19, USP25, OTUD3, OTUD4 and YOD1. Studies have reported that TRIM44 and OTUD4 can suppress the K48-linked polyubiquitylation of MAVS in response to virus infection (42, 43). USP19, USP25, OTUD3 and YOD1 are reported to interact with MAVS and deubiquitinates K63-linked ubiquitinated MAVS for negative regulation of type I IFN signaling (44–47).

**Table 1:** Protein modifications regulate the MAVS signaling pathway.

Post-translational modification of proteins	Regulatory factor	References
K27-linked ubiquitylation	MARCH8 RNF34 RNF5	(23–27)
	TRIM21	(28, 29)
K48-linked ubiquitylation	TRIM25、TRIM28 MARCH5、RNF5 RNF90、RNF115 RNF146、AIP4 SUMRF1/2	(30–38)
	TRIM44、OTUD4	(42, 43)
K63-linked ubiquitylation	TRIM 31	(40)
	N4BP3	(41)
Deubiquitination	USP19、USP25 OTUD3 、YOD1	(44–47)
	TRIM44、OTUD4	(42, 43)
Phosphorylation	c-Abl	(49)
	NLK	(50)
Dephosphorylation	PPM1A	(52)
	PPM1G	(53)

### 3.3 Phosphorylation and dephosphorylation of MAVS

Phosphorylation and dephosphorylation are equally critical in antiviral innate immunity.

Recent research has demonstrated that TBK1 directly targets MAVS, playing a crucial role in activating IRF3 (48). The non-receptor tyrosine kinase c-Abl positively regulates the RLR signaling pathway by phosphorylation of Y9, Y30 and Y71 in the CARD domain of MAVS (49). Studies have shown that Nemo-like kinase (NLK) interacts with MAVS during the latter stages of viral infection, resulting in MAVS phosphorylation, degradation, and subsequent inactivation of IRF3 (50). Conversely, dephosphorylation of MAVS can also act as a switch to deregulate the body’s antiviral signaling (51). Purified PPM1A completely eliminated phosphorylation on MAVS, indicating that PPM1A directly dephosphorylated phospho-MAVS Protein phosphatase 1A (Phospha-tase magnesium-dependent 1A, PPM1A) and protein phosphatase 1G (Phosphatase magnesium-dependent 1G, PPM1G) function as de-phosphatases, both of which can dephosphorylate MAVS and subsequently silence the RLR antiviral signaling pathway (52, 53). All the related modifications of MAVS are listed in **Table 1**.

## 4 MAVS in cardiac disease

**Figure 4:**
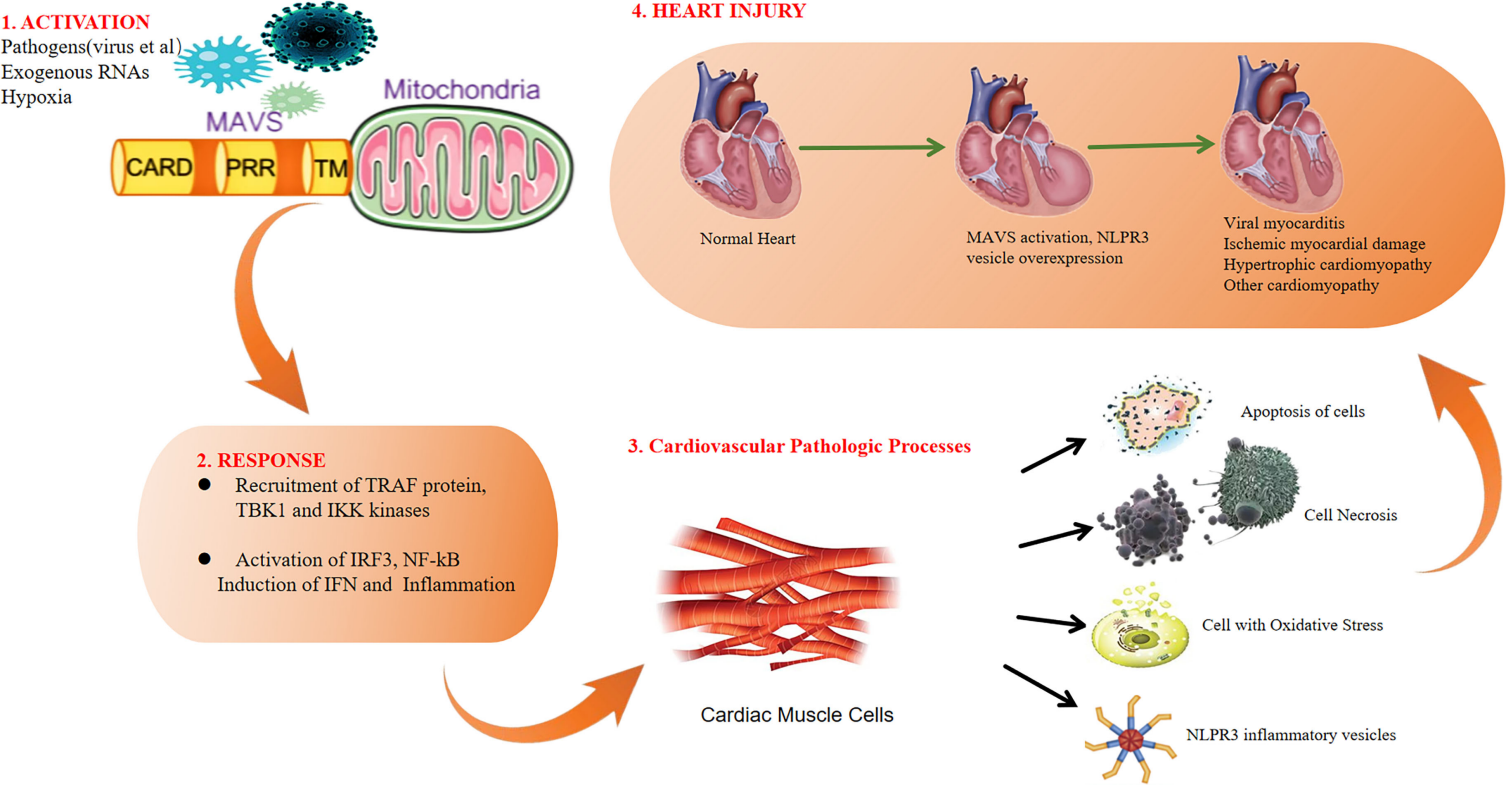
Overview of MAVS roles in heart diseases.

Emerging evidence indicates that MAVS is intricately involved in the pathogenesis of heart diseases. This review highlights recent progress in understanding the contributions of MAVS in viral myocarditis, ischemic myocardial damage, hypertrophic cardiomyopathy and other cardiomyopathy. The roles of MAVS in heart diseases are summarized in **Figure 4**.

### 4.1 MAVS in viral myocarditis

Myocarditis is defined as inflammation of cardiac tissue resulting from an inflammatory infiltrate with or without myocyte necrosis. There are experimental studies showing that activation of cardiac inflammation causes left ventricular remodeling and left ventricular dysfunction. Viral myocarditis presents a substantial risk of sudden death in young individuals due to the heart’s limited ability to regenerate damaged cardiomyocytes. The disease involves three stages: viral infection, autoimmune, and remodeling of dilated cardiopathy and then leads to cardiac failure (54, 55). Some studies show that type I interferon treatment plays an effective therapeutic for viral myocarditis, with cardiac myocytes expressing higher levels of type I interferon compared to cardiac fibroblasts (56–59). MAVS, as a critical adapter for type I interferon expression, has been reported to be essential for high levels of type I interferon expression in cardiac myocytes (14). Maria G. et al. furtherly finds that MAVS signaling is essential for cardiac clearance of the virus. In the absence of MAVS signaling, persistent infection leads to focal myocarditis and vasculitis. MAVS knockout mice were infected with Chikungunya virus (CHIKV) and found that the MAVS was essential for the clearance of CHIKV infection (12). Coxsackie B viruses (CVB) are enteroviruses commonly linked to myocarditis. Jennifer P et al. found that MAVS was critical for type I interferon responses to CVB, as the lack of MAVS results in the absence of type I interferon production and mortality in mice infected with CVB (60). Coxsackievirus B3 (CVB3) is a common enterovirus and CVB3 infection induces mitophagy and then suppresses IFN pathways, and MAVS is involved in this process (61). Moreover, TRIM18 and TRIM21 serve as regulator of IFN-β signaling by targeting MAVS during CVB3 infection. Loss of TRIM18 enhances production of type I IFN and shields mice from viral myocarditis. Mechanistically, TRIM18 recruits protein phosphatase 1A (PPM1A) to dephosphorylate TBK1, inhibiting the interaction of TBK1 with MAVS, thereby dampening antiviral signaling (13). However, TRIM21 catalyzes the K27-linked polyubiquitination of MAVS, and enhances type I interferon signaling and consequently reducing CVB3 viral replication (62). Encephalomyocarditis virus (EMCV) is a zoonotic pathogen known to causes myocarditis. A study found EMCV VP2 acted as a negative regulator of the IFN-β pathway, and the structural protein VP2 interacted with MAVS to block the type I interferon signaling (63). Another research found metalloproteinase domain 9 (ADAM9) bind to MDA5 and promoted (MAVS), and thereby induced type I interferon production during encephalomyocarditis virus infection, which provides a therapeutic target for viral myocarditis (64).

### 4.2 MAVS in Ischemic myocardial injury

Ischemic myocardial injury is a common cardiovascular emergency and leads to higher morbidity and mortality. Despite therapeutic advancements, Ischemic cardiomyopathy remains a significant public health challenge, with 1-year mortality at 16% and 5-year mortality approaching 40% in the USA and Europe (65, 66). Increasing evidence showed that inflammasome activation and apoptosis were participated in the pathogenesis of ischemic myocardial injury. NLRP3 is a key component of the inflammasome. MAVS is essential for NLRP3 inflammasome activity (67). Following myocardial infarction, NLRP3 inflammatory vesicles are upregulated, potentially contributing to the progression of infarct size during ischemia-reperfusion (68). Study finds that inhibiting the NLRP3 inflammasome reduces infarct size and preserves cardiac function in an animal model of MI (69). Recent study found TAX1BP1 exerted cardioprotective effects in acute myocardial infarction by inhibiting inflammasome activation in an RNF34/MAVS-dependent mechanism (70). Another study showed that NLRX1 played a protectional role in myocardial ischemic injury by suppressing MAVS-dependent inflammation and apoptosis (71). Inflammasome activation and pyroptosis are reported to contribute to the pathogenesis of myocardial ischemia-reperfusion (I/R) injury. In the context of ischemia-reperfusion (I/R) injury, research demonstrated an increase in levels of the E3 ubiquitin ligase membrane-associated RING finger protein 2 (MARCH2) in ischemic hearts (72). Interestingly, the absence of MARCH2 worsened myocardial infarction and cardiac dysfunction. Moreover, MARCH2 played a protective role against cardiomyocyte pyroptosis and myocardial injury during ischemia-reperfusion by negatively regulating the PGAM5/MAVS/NLRP3 pathway (72). These findings align with Stefano et al.’s study, which showed that inhibiting NLRP3 pharmacologically in the hippocampus limited secondary inflammatory damage and reduced infarct size one hour after myocardial ischemia-reperfusion in mice (73).

### 4.3 MAVS in hypertrophic cardiomyopathy

Belonging to the nucleotide-binding oligomerization domain (NOD)-like receptor family, NLRP3 is associated with cardiac inflammation (74). MAVS, on the other hand, is essential for maximizing the function of NLRP3 inflammatory bodies and contributes significantly to regulating inflammation (48). In their study, Li et al. observed that the absence of NLRP3 hastened cardiac hypertrophy, fibrosis, inflammatory reactions, and worsened cardiac function in a mouse model of pressure overload-induced cardiac remodeling (75). These results suggest that targeting NLRP3 could hold therapeutic promise for managing cardiac remodeling and heart failure (76). Jing Zong et al. observed upregulated NOD2 expression in cardiomyocytes of aortic fasciculation-type hypertrophic mice (77). They also demonstrate that NOD2 inhibits myocardial hypertrophy and fibrosis in mice, counteracting hypertrophic stimuli by inhibiting TLR4 and TGF-b/Smad signaling pathways, while regulating pro-fibrotic cytokines and collagen content (77). However, recent studies have revealed that MAVS acts nucleotide-binding oligomeric structural domain-containing protein 1/receptor-interacting protein 2 (NOD1/RIP2) downstream to promote cardiac hypertrophy in response to transecting pressure overload induced by aortic constriction (TAC). Nod1^-/-^ and RIP2^-/-^ mice demonstrated better survival, enhanced cardiac function, and reduced cardiac hypertrophy during subjecting to TAC. This process critically involves MAVS regulation in the inflammatory response, and mitochondrial energy metabolism. The NOD1/RIP2/MAVS signaling complex effectively coordinates remodeling, inflammatory response and mitochondrial energy metabolism in stressed cardiomyocytes (78).

### 4.4 MAVS in other cardiomyopathy

Mitochondria plays a crucial role in energy production. MAVS, located on the mitochondrial outer membrane, regulates mitochondrial dynamics, energetics and facilitates the interaction of RLR signaling and glucose metabolism (79). Recent research revealed that silencing MAVS mitigated the radiation-induced mitochondrial dysfunction (including mitochondrial membrane potential disruption and ATP production) (80). MAVS suppression affects both mitochondrial function and morphology in cardiomyocytes (78). Qian Wang et al. found that MAVS deficiency exacerbated the deterioration of cardiac insufficiency and cardiac dilation. Metabonomic suggested MAVS deletion disturbed energy metabolism, especially lipid metabolism. Knockout of MAVS induced the mitochondrial structure and function impairments, leading to elevated mitochondrial ROS levels (11).

Although the role of MAVS in cardiac diseases requires further explored, its significance and relevance to cardiac diseases are widely acknowledged. Consequently, it is imperative to explore and clarify its mechanism of action in cardiac diseases.

## 5 MAVS/NLRP3 in cardiovascular diseases

NLRP3 is a crucial component of the inflammasome, involved in regulating inflammatory responses within the immune system. Emerging evidence has indicated that the NLRP3 inflammasome plays an important role in cardiovascular diseases, such as atherosclerosis, ischemic heart disease, dilated cardiomyopathy, hypertensive heart disease, metabolic disorders and diabetic cardiomyopathy, cancer therapy-associated cardiac injury, myocarditis, and pericarditis (74). MAVS mediates recruitment of NLRP3 to mitochondria, and activates of the NLRP3 inflammasome *in vivo (* 67). Peter Duewell et al. first demonstrated the role of the NLRP3 inflammasome in promoting atherosclerosis in western diet-fed LDL receptor-deficient mice (81). Negatively regulating the MAVS/NLRP3 pathway played a protective role against cardiomyocyte pyroptosis and myocardial injury during ischemia-reperfusion (70, 72). Studies reported that inhibiting NLRP3 and other inflammasome components in animal model of ischemic cardiac injury showed beneficial effects in terms of reduced infarct size and improved cardiac function (82, 83). Several NLRP3 inflammasome inhibitors are developing for CVD in preclinical and clinical stage (**Table 2**). Some of these inhibitors block the NLRP3 inflammasome, others block NLRP3 signaling. Overall, the investigation of the close association between MAVS and the NLRP3 inflammasome in cardiovascular diseases provides us with deeper insights into the immune mechanisms underlying these conditions, while also offering potential drugs targeted NLRP3 for future therapeutic strategies.

**Table 2:** NLRP3 inhibitors under clinical development in cardiovascular diseases: name and chemical structure.

Name	Chemical structure
BAY 11-70820	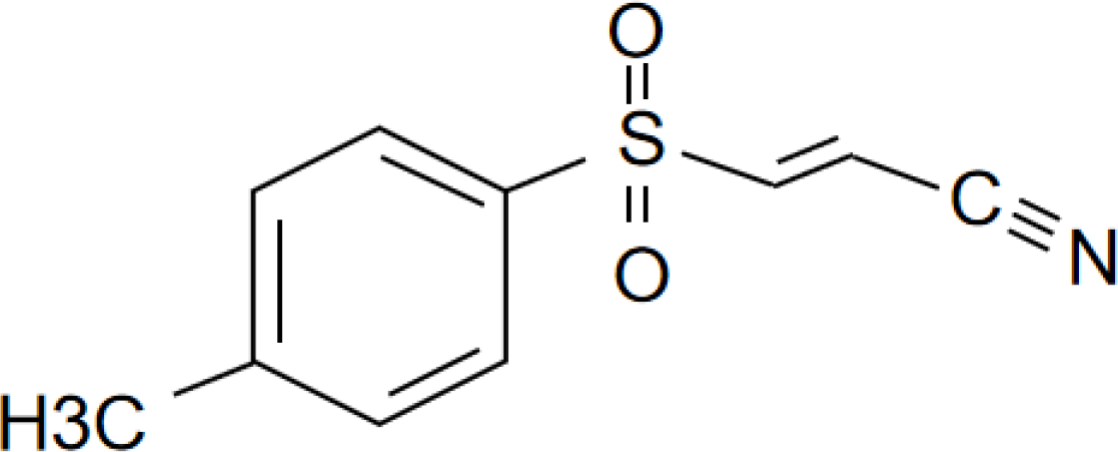
INF4E	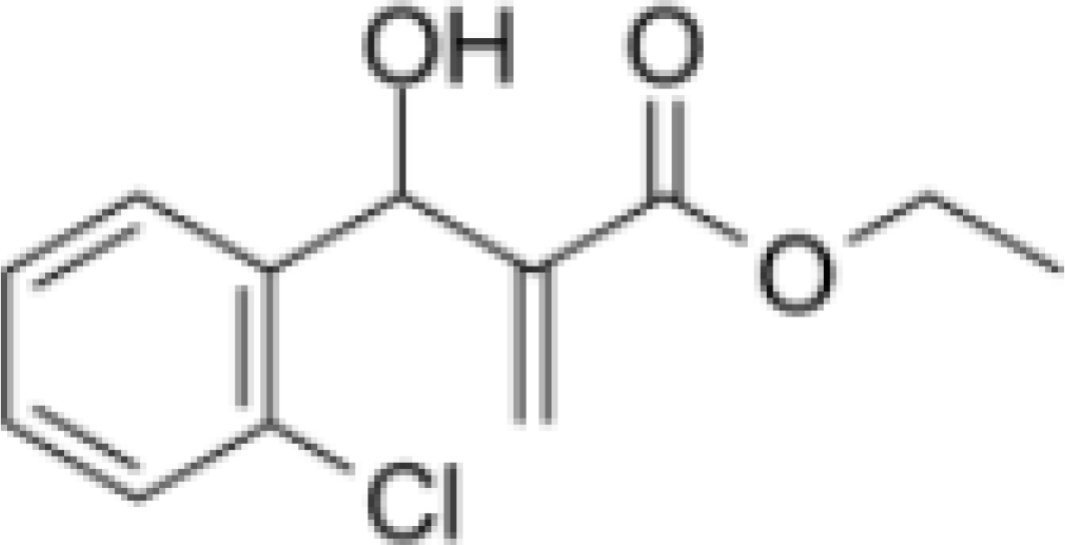
OLT1177	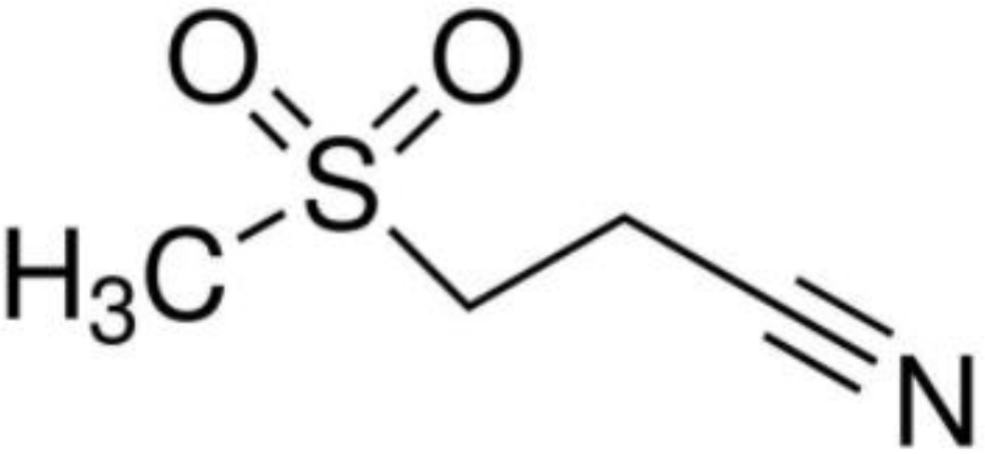
Tranilast	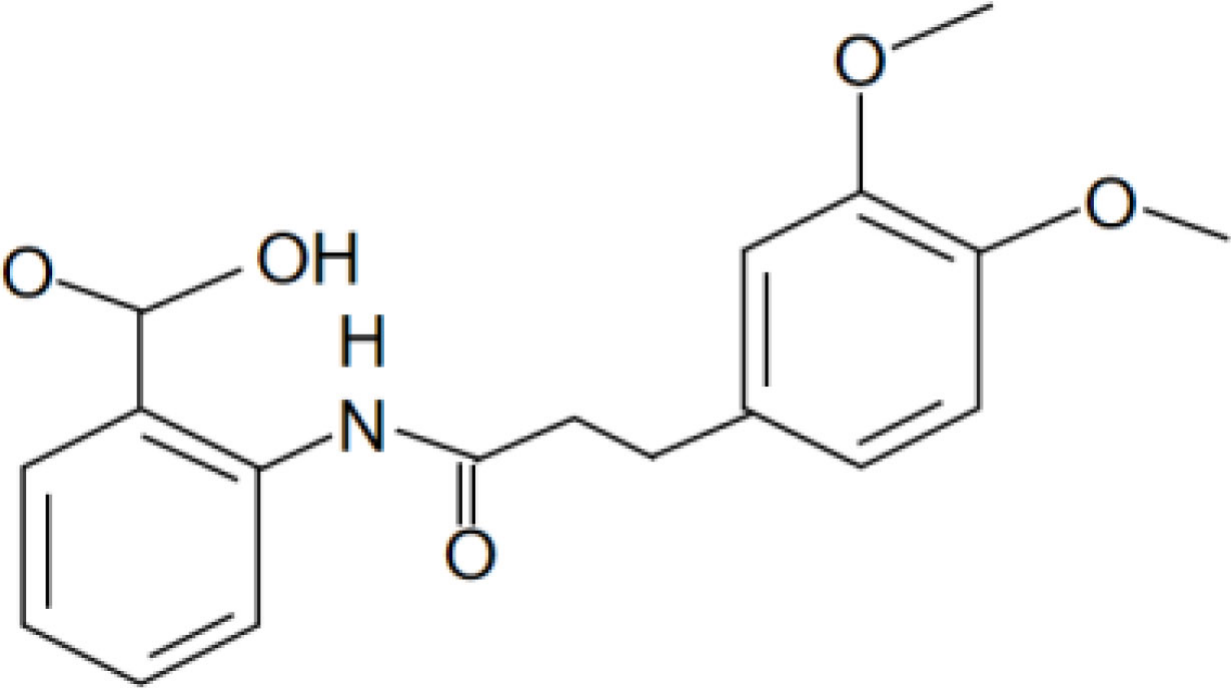
CY-09	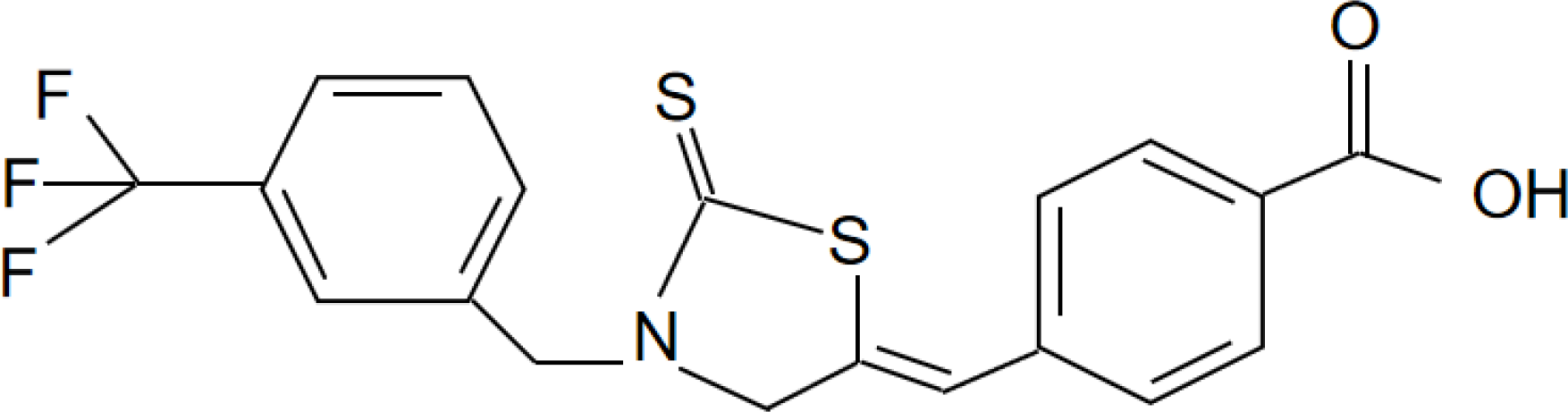
MCC950	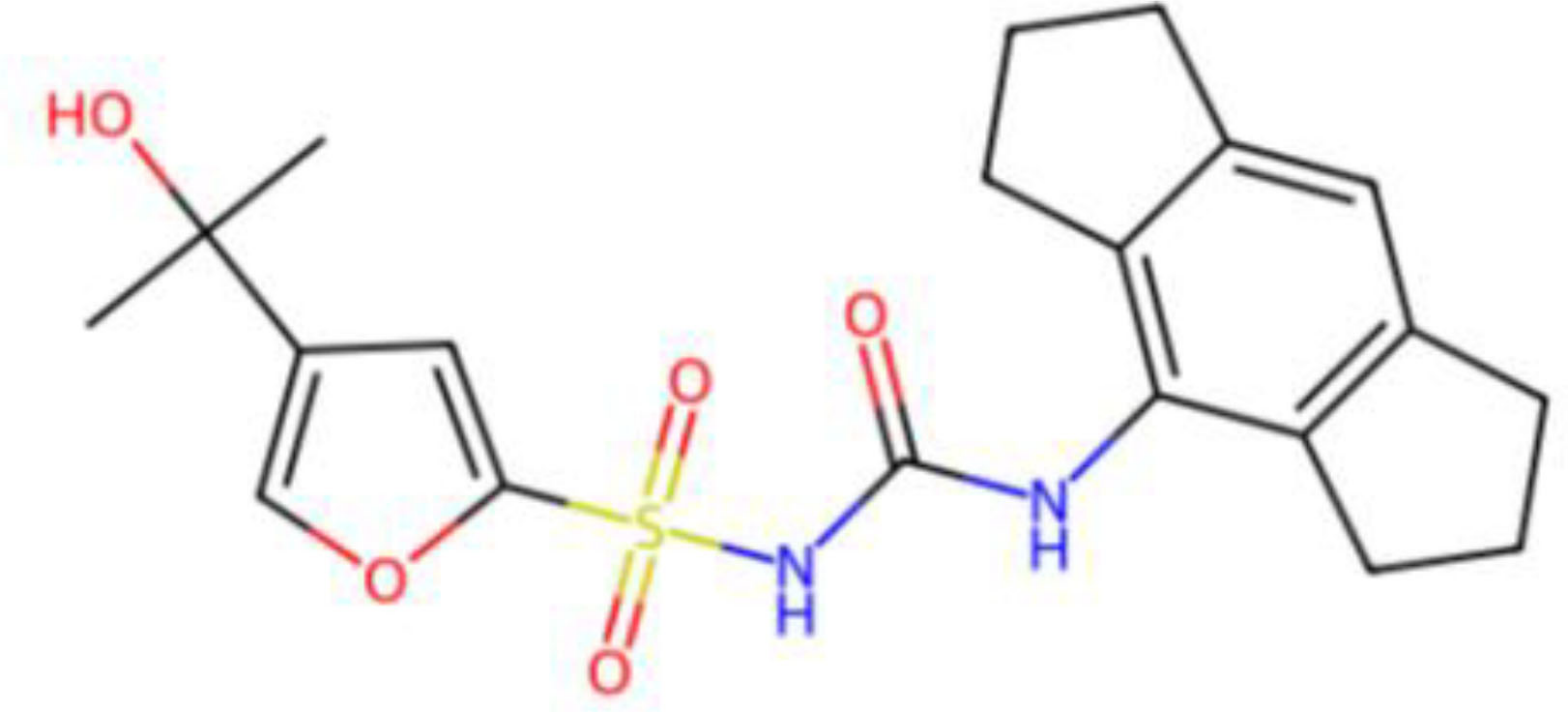
16673-34-0	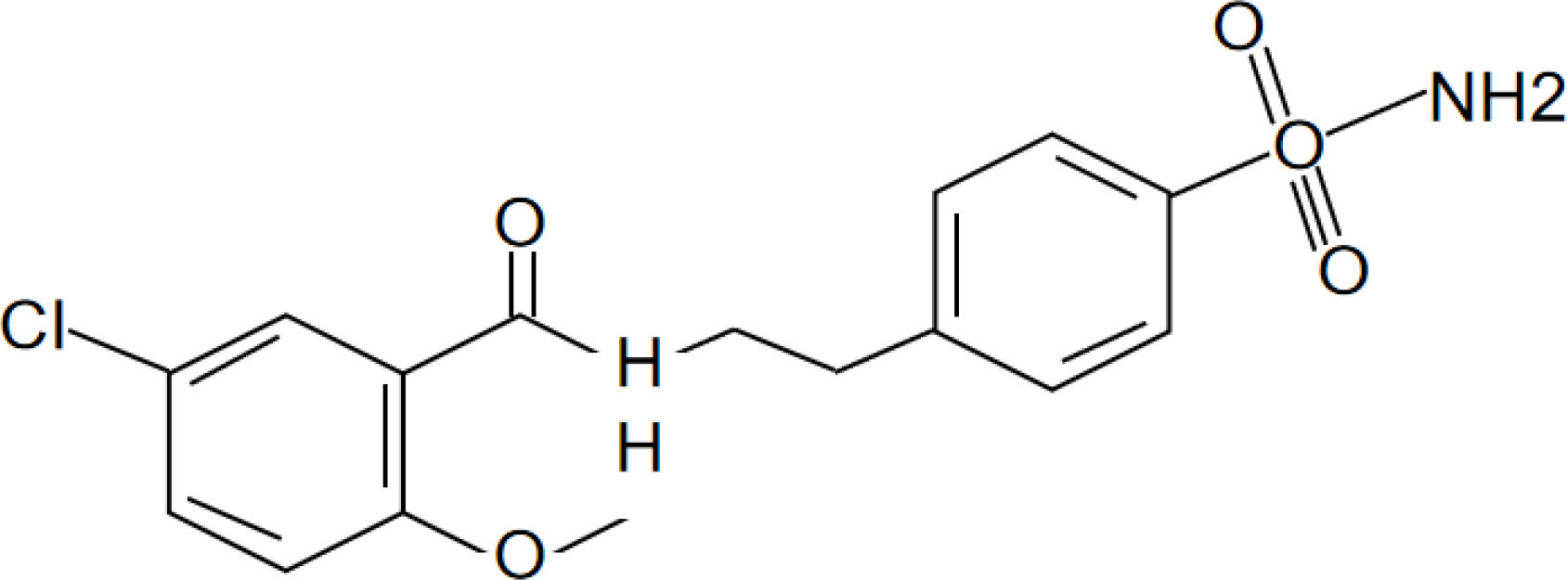

## 6 Conclusion and perspectives

Cardiovascular disease (CVD) poses a substantial public health burden, and is currently a leading cause of disability and mortality among the elderly. The epidemiology of CVD has shifted from predominantly affecting developed countries to becoming a global disease, with the highest prevalence rate in our country. CVD and its associated complications are the primary cause of mortality in patients. MAVS, a crucial connector protein in the RLR signaling pathway, significantly contributes to the pathology of both innate immunity and cardiac diseases. Nevertheless, the regulatory mechanisms of MAVS-mediated antiviral signaling pathways in various animal organisms remain unclear to date. Studying the regulatory mechanisms of MAVS in cardiovascular diseases is anticipated to provide some insights for identifying relevant target drugs against this pathway and treating related diseases. Furthermore, MAVS plays a critical role in enhancing optimal NLRP3 inflammasome activation. Specifically, MAVS promotes NLRP3 oligomerization by recruiting it in proximity to mitochondrial ROS, a crucial element for NLRP 3 activation. Several studies suggest that MAVS and its downstream factor NLRP 3 may provide promising strategies for treating heart disease. Additionally, TRIM29 targets MAVS to negatively regulate the production of antiviral type I interferons and the activation of inflammasomes, thereby modulating the host immune response to viral infections (84, 85). Junying Wang et al. found that the deficiency of TRIM29 alleviated viral myocarditis (86). Similarly, some studies reported that TRIM18 regulates the TBK1 and MAVS signaling pathways by recruiting protein phosphatase 1A (PPM1A). Deletion of TRIM18 protects mice from viral myocarditis (13, 52). Therefore, targeting TRIM29 and TRIM18 may provide new therapeutic approaches for reducing myocardial inflammation and improving cardiac function by regulating the MAVS signaling pathway.

Cardiovascular diseases are closely associated with the immune system. Immune responses and microenvironment play a pivotal role in the initiation, progression, and prognosis of cardiovascular diseases. A recent study showed that M1-like pro-inflammatory macrophages also contributed to myocardial injury by secreting pro-inflammatory exosomes and pro-inflammatory miRNAs which inhibited angiogenesis and cardiac healing (87). Li Liu et al. found that neutrophils can release annexin A1 and lactoferrin, as well as engage in chemokine scavenging, thereby halting the migration of granulocytes into the infarcted myocardial tissue (88). Depletion of neutrophils was linked to an exacerbation of fibrosis and a deterioration in heart function in the chronic MI model (89). Moreover, proinflammatory cytokines play a critical role in the pathogenesis of heart failure. Douglas L. Mann proposed that inflammatory mediators such as TNF-α, IL-1β, and IL-6 are remarkable (90).

Recent studies have revealed the role of MAVS in immune cell infiltration and activation, particularly in macrophages, neutrophils, and T cells, which play key roles in the immune response in cardiovascular diseases. Macrophages, as important immune cells in cardiovascular diseases, directly influence disease progression through their polarization. Specifically, activation of MAVS can promote M1 macrophage polarization by enhancing the secretion of type I interferons, such as IFN-β. M1 macrophages not only amplify local inflammation by releasing pro-inflammatory cytokines such as TNF-α and IL-1β but also promote immune cell infiltration and tissue damage in cardiovascular pathologies such as atherosclerosis, myocardial infarction and cardiac remodeling (91, 92). Neutrophil infiltration and activation are also crucial in driving cardiac inflammation and damage. Freja et al. demonstrated that in RSV-infected mice, the MAVS signaling pathway is essential for neutrophil recruitment and activation through type I interferon production (93). Moreover, the involvement of T cells in chronic cardiovascular diseases has become increasingly significant. For instance, Huanle Luo et al. investigated the role of MAVS in regulating host immunity against the live attenuated West Nile virus (WNV) vaccine strain. They found that MAVS is critical for enhancing the primary CD4 T cell response during NS4B-P38G vaccination (94). In summary, the excessive activation and dysregulation of these immune cells drive the progression of inflammatory responses and exacerbate tissue damage in cardiovascular diseases. Future studies should further explore the specific mechanisms of MAVS as an immune modulator in cardiovascular diseases, providing new insights for immune-based therapies in cardiovascular disorders.

However, the existing body of literature primarily focuses on animal or cellular models of heart disease, neglecting the exploration of MAVS expression levels in clinical patients. Therefore, attaining a comprehensive understanding of the regulatory mechanisms involved in this pathway is of utmost importance as it could serve as a guiding principle for the development of innovative therapeutic strategies to address cardiac diseases in the future.
